# The Influence of Diurnal Temperature Variation on Degree-Day Accumulation and Insect Life History

**DOI:** 10.1371/journal.pone.0120772

**Published:** 2015-03-19

**Authors:** Shi Chen, Shelby J. Fleischer, Michael C. Saunders, Matthew B. Thomas

**Affiliations:** 1 Department of Population Health and Pathobiology, North Carolina State University, Raleigh, North Carolina, United States of America; 2 National Institute for Mathematical and Biological Synthesis, Knoxville, Tennessee, United States of America; 3 Department of Entomology, Pennsylvania State University, University Park, Pennsylvania, United States of America; 4 Center for Infectious Disease Dynamics, Pennsylvania State University, University Park, Pennsylvania, United States of America; USDA-ARS, UNITED STATES

## Abstract

Ectotherms, such as insects, experience non-constant temperatures in nature. Daily mean temperatures can be derived from the daily maximum and minimum temperatures. However, the converse is not true and environments with the same mean temperature can exhibit very different diurnal temperate ranges. Here we apply a degree-day model for development of the grape berry moth (*Paralobesia viteana*, a significant vineyard pest in the northeastern USA) to investigate how different diurnal temperature range conditions can influence degree-day accumulation and, hence, insect life history. We first consider changes in diurnal temperature range independent of changes in mean temperatures. We then investigate grape berry moth life history under potential climate change conditions, increasing mean temperature via variable patterns of change to diurnal temperature range. We predict that diurnal temperature range change can substantially alter insect life history. Altering diurnal temperature range independent of the mean temperature can affect development rate and voltinism, with the magnitude of the effects dependent on whether changes occur to the daily minimum temperature (*Tmin*), daily maximum temperature (*Tmax*), or both. Allowing for an increase in mean temperature produces more marked effects on life history but, again, the patterns and magnitude depend on the nature of the change to diurnal temperature range together with the starting conditions in the local environment. The study highlights the importance of characterizing the influence of diurnal temperature range in addition to mean temperature alone.

## Introduction

Climate warming is expected to influence the dynamics and distribution of terrestrial ectotherms, such as insects, because their physiology and ecology are strongly dependent on ambient temperature [1–8]. While research has focused on the potential effects of changes in mean temperature at relatively coarse temporal scales (e.g. seasonal or yearly mean temperature) [6], recent studies have demonstrated that temperature variation at much finer scales can also influence insect life history [9–14]. These studies have highlighted the importance of considering diurnal temperature range (DTR thereafter) along with mean daily temperature in order to fully understand the consequences of climate change.

DTR is the range between daily maximum (*Tmax*) and minimum temperature (*Tmin*). How DTR is likely to change under current climate change projections is not clear [15–16]. There is evidence to suggest that *Tmax* will increase while *Tmin* decreases, or that *Tmax* will increase at a faster pace than *Tmin*, in both cases increasing the DTR [17–18]. On the other hand, there is also evidence to support greater increases in *Tmin* relative to *Tmax*, resulting in smaller DTRs [19–22]. Given these contrasting scenarios, there is a need to comprehensively and systematically investigate how changes in DTR could impact insect life history.

We use the grape berry moth (*Paralobesia viteana* Clemens, Lepidoptera: Tortricidae) as a model organism. *P*. *viteana* is a significant vineyard pest in the United States, and its life history has been well characterized [23–28]. In this study, we first demonstrate how degree-day accumulation changes under the same mean temperature with various DTR conditions. We then contrast how key aspects *P*. *viteana* life history (i.e. voltinism and mean emergence/eclosion time from pupa in each generation) change under the same mean temperature but with various DTR conditions using a published degree-day model for *P*. *viteana* [28]. Finally, we demonstrate and contrast how *P*. *viteana* life history changes under the same increased mean temperature with various DTR conditions and assess the implications of climate change on insect life history.

## Materials and Methods

### Diurnal Temperature Range (DTR) Change and Degree Day Accumulation

The diurnal temperature profile could be extrapolated accurately from daily maximum and minimum temperature as well as photoperiod using a modified sine curve model [29–30]. DTR was defined as the difference between daily maximum temperature (*Tmax*) and minimum temperature (*Tmin*). The degree-day accumulated in a given day was defined as the total heat unit accumulated (the area under the temperature curve) above the developmental threshold (*Tbase*) of the insect over the entire day, where *Tbase* was estimated from a linear regression of development rate against temperature [31]. Consequently, using mean temperature (*Tmean*) alone without considering the DTR could yield a different degree-day accumulation. More specifically, if *Tmean* was less than *Tbase*, then the insect accumulated zero degree-days in that day when a degree day model without DTR information is used; while if DTR was included, the insect accumulated some heat units, given that the daily maximum temperature (*Tmax*) is above *Tbase*. In general, the degree-day accumulation in the given time period (e.g. a day) was computed as the area of the actual temperature curve above the *Tbase* (base development temperature threshold). If *Tbase* was above *Tmax* (daily maximum temperature), then the degree-day accumulation was zero and there was no effect from the DTR. Similarly, if *Tbase* was below *Tmin* (daily minimum temperature), there was no effect from considering DTR when estimating degree-day accumulation, because the degree-day accumulation was always *Tmean-Tbase* (assuming *Tmean*, daily mean temperature, remained the same). Hence, DTR was an important factor for estimating degree-day accumulations when *Tbase* fell between *Tmax* and *Tmin*. The *Tmax* and *Tmin* (and also latitude information of the site) were used to extrapolate hourly temperature profile in a day based on the modified sine-curve method [29–30]. In each day, the starting diurnal temperature profile was constructed from 10-year averaged (starting) *Tmax* and *Tmin* (*Tmax* and *Tmin* were both averaged from 10 observations to eliminate any potential outlier temperatures) under the same *Tmean* with different DTR conditions.

To simulate various DTR change conditions, we used a parameter *k* to adjust the DTR while keeping the daily mean temperature the same as the current condition (i.e., the DTR using the modified sine curve applied to observed *Tmax* and *Tmin*, see below). The new daily maximum temperature (*T’max*) was computed as the starting *Tmax* plus a fraction of the starting daily mean temperature (*kTmean*). Similarly the altered daily minimum temperature (*T’min*) was the starting *Tmin* minus the same fraction of the starting daily mean temperature (*kTmean*). The difference between new DTR and current DTR is 2*kTmean*.

We allowed *k* to vary from −0.4 to 0.4 with 0.1 increments, hence there were a total of nine conditions (one with the current *k* = 0, plus eight with a DTR change when *k≠*0). When the same *k* is applied to *Tmin* and *Tmax*, the DTR changes but *Tmean* is held constant. Four conditions represent an increasing DTR (when *k*>0) and four conditions represent a decreasing DTR (*k*<0). For instance, *k* = 0.1 results in 1°C increase in *Tmax* and a simultaneously 1°C decrease in *Tmin* (assuming *Tmean* = 10°C, with a total of 2°C change of DTR), and *k* = 0.4 yields a 4°C increase in *Tmax* with 4°C decrease in *Tmin* (with 8°C change of DTR); in both cases *Tmean* = 10°C. Daily maximum and minimum temperatures from 2001 to 2010 in Erie, Pennsylvania (42.13°N, 80.09°W, in the Lake Erie coastal plain) and Stuarts Draft, Virginia (38.03°N, 79.03°W, in the Blue Ridge Mountains valley), both locations of major grape production in the USA and with *P*. *viteana* infestation, were acquired from National Oceanic and Atmospheric Administration, USA. In general, both locations had similar annual mean temperature (10.17°C and 9.86°C for Erie and Stuarts Draft, respectively), with Stuarts Draft experiencing colder winters and earlier springs (and larger annually temperature fluctuations). Their latitudes were used to calculate photoperiod, another major environmental factor that determined the moth’s life history. Unlike temperature, photoperiod in the same calendar day at a given location did not change from year to year. *Tmax* and *Tmin* for each day were averaged across these 10 years, adjusted by the method mentioned above to simulate various DTR conditions, and used to calculate degree-day accumulations. Although the annual mean temperature only had minor fluctuations for both locations in the recent 10 years (±1°C), the temperature range could change more than 3°C during the same period. The degree-day differences under these conditions in these two locations at selected days of the year were computed and compared.

### Grape Berry Moth Life History Change under the Same Mean Temperature Conditions (with Various DTRs)

The Grape Berry Moth (*Paralobesia vetiana* Clemens) is a significant vineyard pest in the U.S. Its life history has been comprehensively studied. The processes of diapause termination, development from egg-to-adult under non-diapausing conditions, and diapause induction have been modeled as function of temperature and photoperiod [23–26]. The base developmental threshold temperature for *P*. *viteana* (*Tbase*) was reported as 8.4°C [24,26] and the simulation started from day 1 in the year (Jan. 1^st^) through the end of the year (Dec. 31^st^). The life history of *P*. *viteana* is determined by the complicated nonlinear interaction between temperature and photoperiod. During spring, overwintering pupae from the previous year (the 1^st^ generation) broke diapause and emerged by accumulating about 210 degree-days (DD) on average, starting from Jan. 1^st^, and laid eggs. The successive generations (from 2^nd^ generation and thereafter) required an average of 484 DD to complete the entire life history (from egg to larva, pupa, and until reproductively mature adult, under non-diapause condition). Diapause was mediated by photoperiod (and not temperature) experienced by the egg after summer solstice. If an individual was determined to diapause then the larva developed to pupa stage only and not to further adult stage in current year, and consequently did not count as a complete generation in current year. A more detailed complete life history individual-based simulation model was developed and validated [28] and provided as S1 Appendix, and all the codes were written in *R* [32].

We applied this modeling framework for *P*. *viteana* [28] and contrasted modeled output for the eight conditions reflecting DTR changes to the output without a change in DTR (where *k* = 0, defined as the current condition). We ran the *P*. *viteana* life history simulation at Erie, PA and Stuarts Draft, VA under various DTR change conditions described in the previous section (a total of 9 conditions including the current condition) and reported the mean voltinism (number of generations per year, averaged from 1,000 simulations), mean adult emergence time of each generation, and duration of each generation, for each of these conditions. The pairwise differences of emergence time among the 9 conditions in each generation (2 through 4) were tested by the Tukey honest significance difference (HSD) analysis. The number of generation was regressed against the DTRs (*k* value) to test whether the slope was significantly different from 0. The mean durations of each generation were also regressed against the DTRs (*k* value). The detailed modeling and calculation of *P*. *viteana* voltinism is provided in S1 Appendix.

### Grape Berry Moth Life History Change under Increasing Mean Temperature Conditions (with Various DTRs)

As shown in the results, *P*. *viteana* life history changed substantially under changing DTR conditions even when *Tmean* remained the same (Tables 1 and 2). Next we investigated how changing DTR along with changing *Tmean* has the further potential impact on insect life history. Similar to the previous approach, we now used two parameters, *k*_*1*_ and *k*_*2*_, to modify *Tmin* and *Tmax*, respectively. The modified daily minimum temperature (*T’min*) and maximum temperature (*T’max*) were computed as below, with change in mean temperature approximately 0.5(*k*_*1*_-*k*_*2*_) *Tmean* and change of DTR as −(*k*_*1*_ *+k*_*2*_) *Tmean;* consequently, change in mean temperature is not independent from change of DTR:

$$\begin{array}{c} T^{\prime }min=Tmin+k_{1}\overset{-}{T}\\ T^{\prime }max=Tmax+k_{2}\overset{-}{T} \end{array}$$

Thus, positive *k*_*1*_ values lead to higher nighttime temperatures (higher *Tmin*), but negative *k*_*2*_ values leads to higher daytime temperatures (higher *Tmax*).

**Table 1:** Degree-day Accumulation at Different Locations in Different Days in a Year under Same Mean Temperature with Various DTR Conditions.

	Day 50		Day 100		Day 150		Day 200	
*k*	Erie	Stuarts	Erie	Stuarts	Erie	Stuarts	Erie	Stuarts
−0.4	0	0	0	0.33	10.06	7.22	15.10	7.41
−0.3	0	0	0	0.53	10.16	7.31	15.32	7.50
−0.2	0	0	0	0.74	10.22	7.40	15.48	7.59
−0.1	0.09	0	0	0.96	10.43	7.50	15.62	7.68
0	0.23	0	0	1.18	10.54	7.61	15.70	7.77
0.1	0.39	0	0	1.39	10.63	7.80	15.95	7.89
0.2	0.55	0	0	1.61	10.86	8.11	16.11	8.07
0.3	0.72	0	0	1.83	10.95	8.50	16.21	8.37
0.4	0.90	0	0.02	2.04	11.15	8.94	16.48	8.77

Note: Stuarts stands for Stuart Draft, VA, in the table. At a given day and location, it is almost consistent that larger DTR (larger *k* value) is related to more degree-day accumulation, even when the mean temperature remains the same. For non-leap years, day 50 is Feb 19, day 100 is Apr 10, day 150 is May 30, and day 200 is Jul 19.

**Table 2:** Mean Duration (Days) of Each Generation at Different Locations under the Same Mean Temperature with Various DTR Conditions.

	2^nd^ Generation		3^rd^ Generation		4^th^ Generation	
*k*	Erie	Stuarts	Erie	Stuarts	Erie	Stuarts
−0.4	44.94	50.83	37.27	37.38	30.85	39.45
−0.3	44.53	50.66	37.26	37.48	32.22	40.89
−0.2	44.16	50.60	37.11	37.60	31.04	39.66
−0.1	43.69	50.38	37.03	38.04	29.35	36.17
0	43.43	49.81	36.78	37.78	28.69	33.95
0.1	43.36	49.48	36.64	37.56	28.60	34.18
0.2	43.04	49.50	36.44	37.74	27.83	34.56
0.3	42.79	49.11	36.21	37.31	27.04	30.42
0.4	42.28	49.19	35.89	37.13	27.36	27.25

Note: Stuarts stands for Stuart Draft, VA in the table. There is consistent trend for larger DTRs (larger *k* values) to result in shorter generation durations for both locations, and this trend is more obvious in the 4^th^ generation at Stuarts Draft, VA. 1^st^ generation is excluded because it is not a complete generation in the current year (i.e. it is the overwintering generation from last year). *k* = 0 represents current condition (see S1 Appendix).

It had been estimated that the annual mean surface temperature will increase by 2–6°C by the end of the 21^st^ century [20,22]. To begin, we comprehensively assigned both *k*_*1*_ and *k*_*2*_ to change from −0.4 to 0.4 (with 0.1 increment, so both *k*_*1*_ and *k*_*2*_ had 9 values, thus 81 conditions bracketed the full range of *k*_*1*_ and *k*_*2*_ values) to bracket this maximum potential increase (and also the maximum decrease—although not likely to occur in the near future, we show it as a demonstration of the influence of DTR on insect life history). We constructed a new daily temperature profile, ran the simulation model, and computed the potential number of generations under a total of these 81 climate change conditions.

Next, we investigated the life history change more realistically for Erie and Stuarts Draft. The annual mean temperature at both locations was approximately 10°C. It was estimated that under mild climate change conditions, Erie would expect an increase of 2°C in mean temperature [28]. However, there was no further deterministic estimation for Stuarts Draft, VA of expected daily mean temperature change under future climates, or any estimate on DTR change for both locations [33–35]. We assumed a minimum change (2°C) as the amplitude of *Tmean* increment in Stuarts Draft, which was the same estimate for Erie, for the end of the 21^st^ century [20,22]. There were multiple configurations of *k*_*1*_ and *k*_*2*_ that achieve an incremental change of 2°C in *Tmean*: *k*_*1*_ to change from 0 to 0.4 while *k*_*2*_ to change from −0.4 to 0 simultaneously (both with 0.1 increment/decrement). Thus, a total of five possible conditions (out of all 81 combinations of climate change) could result in the desired *Tmean* increment, with DTR change as +4, +2, 0, −2, −4°C, respectively. We ran the simulation again and report not only the mean voltinism per year but also generation durations of 2^nd^ to 4^th^ generations at each location. The quantitative influence of both DTR change and *Tmean* change on *P*. *viteana* phenology was tested by a two-factor analysis of variance (ANOVA). Both *k*_*1*_ and *k*_*2*_ were considered as fixed effect and the interaction between *k*_*1*_ and *k*_*2*_ was also included. The model was specified as

μk1k2=μ+αk1+βk2+αβk1k2+ε

where *μ*_*k1k2*_ represented phenology observations (number of generation, each generation duration) and for each *k*_*1*_ and *k*_*2*_ combination, there were 100 replications (i.e. 100 simulations per combination), *μ* was the overall mean, *α*_*k1*_ and *β*_*k2*_ were effects from *k*_*1*_ and *k*_*2*_ respectively, *α β*_*k1k2*_ was the interaction effect, and *ε* was a random error.

## Results

### Diurnal Temperature Range (DTR) Change and Degree Day Accumulation

**Fig 1:**
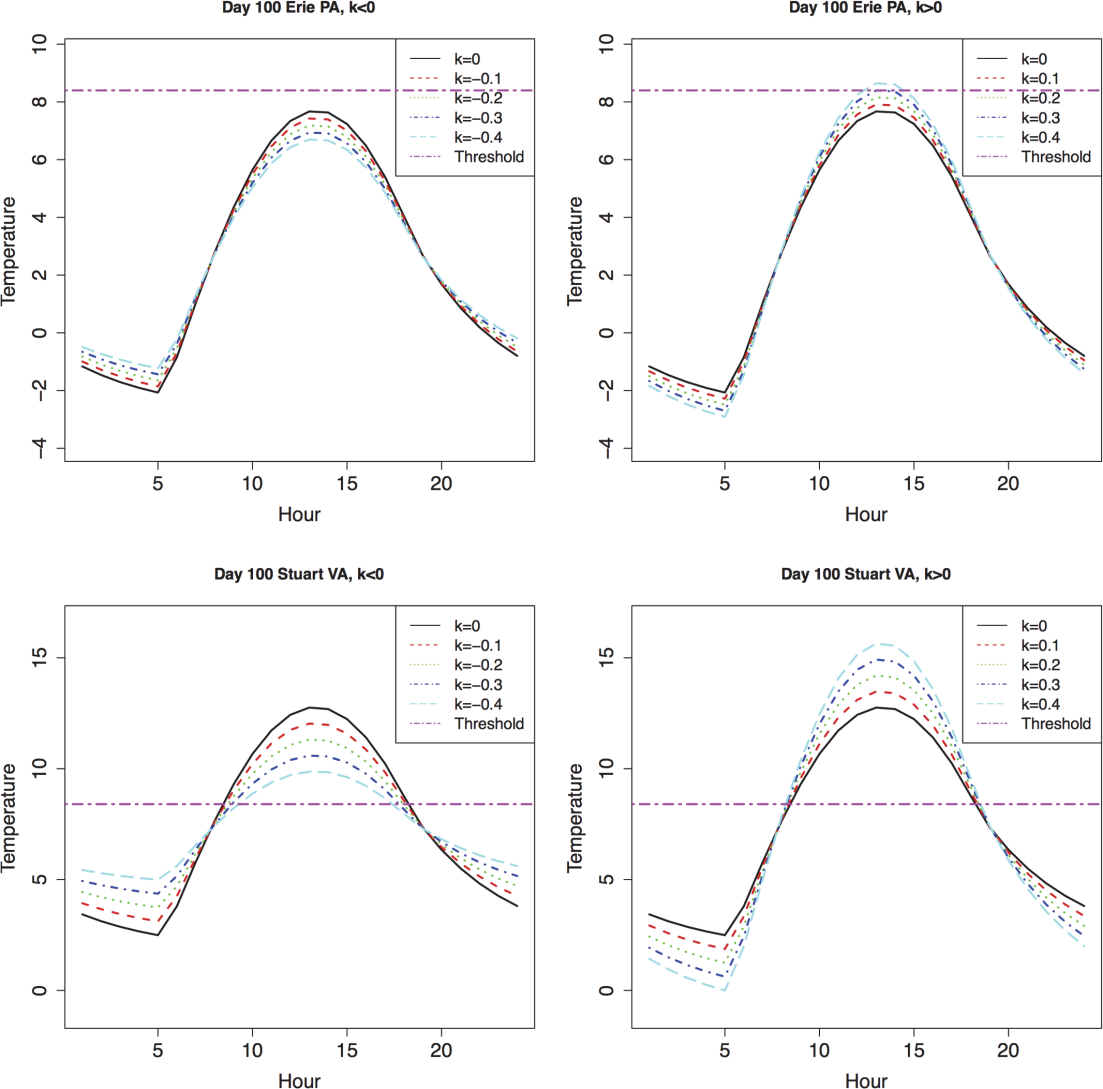
Degree-Days Accumulation at Erie PA and Stuarts Draft VA under the Same Mean with Various DTR Change Conditions. Note: Upper panels: Erie, PA; lower panels: Stuarts Draft, VA. Left panels: decreased DTR conditions (*k*<0); right panels: increased DTR conditions (*k*>0). With same mean temperature, the larger the DTR, the more degree-days can be accumulated in a day.

An example of degree-day accumulation was shown in Fig. 1 for two different locations (Erie, Pennsylvania, and Stuarts Draft, Virginia, USA) on day 100 (Apr. 10 under non-leap year condition). When *k*<0 (decreasing DTR conditions, left two panels in Fig. 1), there was no degree-day accumulation during day 100 at Erie, and degree-day accumulation decreased at Stuarts Draft when *k* was smaller (more negative, indicating smaller DTR). When *k*>0 (increasing DTR conditions, right two panels in Fig. 1), there was a small amount of degree-day accumulation when *k* = 0.4 in Erie (largest DTR condition, and no degree-day accumulation when *k*<0.4). In Stuarts Draft, however, there was substantially more degree-day accumulation under larger *k* value conditions (larger DTR). A more comprehensive degree-day accumulation result under the same *Tmean* with various DTR conditions on selected days of the year (day 50, 100, 150, and 200; Feb 10, Apr 10, May 30, and Jul 19 under non-leap year conditions) for both locations were presented in Table 1. When *Tmax* was below *Tbase* for *P*. *viteana* (8.4°C) before summer solstice (approximately day 50 in Stuarts Draft and day 100 in Erie), there was no degree-day accumulation in any of the 9 DTR conditions. Around and after summer solstice, while *Tmean* remained the same at each location in a given day under these 9 DTR conditions, the actual degree-day accumulations varied substantially. For example, on day 100 at Stuarts Draft, VA, the degree-day accumulation in the largest DTR condition (*k* = 0.4) was 500% more than that in the least DTR condition (*k* = −0.4). Different locations (essentially different latitudes, which were also influential on diurnal temperature profile along with DTR^33^) and different days in the year (seasonality) also influenced degree-day accumulation (Table 1). Consequently, it was not surprising that these differences were able to further impact *P*. *viteana* life history (see below).

### Grape Berry Moth Life History under the Same Mean Temperature Conditions with Various DTRs

**Fig 2:**
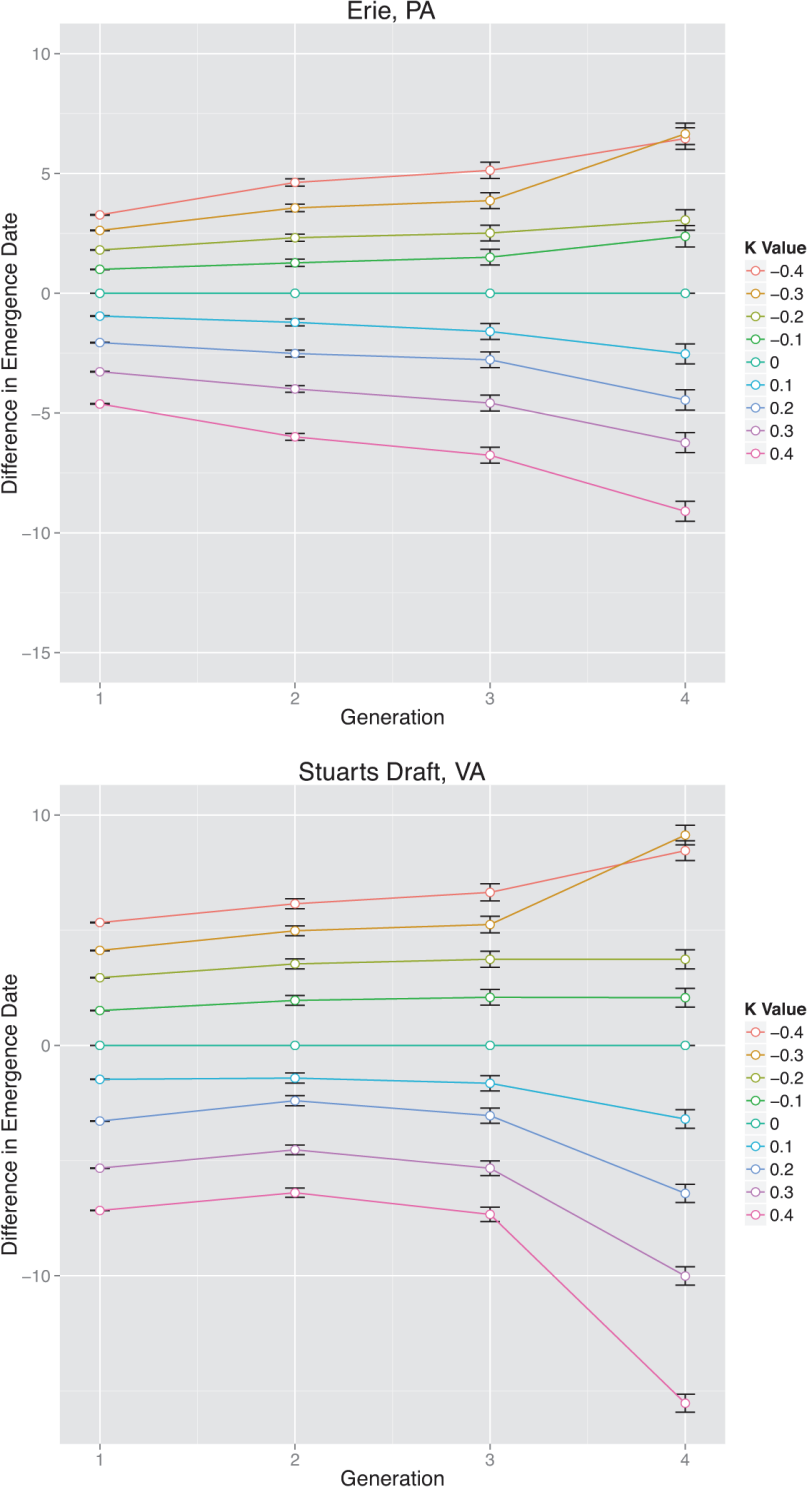
Change in *P*. *viteana* Mean Emergence Date for Four Generations under Same Mean with Various DTR Change Conditions. Note: Upper panels: Erie, PA; lower panels: Stuarts Draft, VA. Different colors indicate different *k* values (see figure legends). The error bars indicate standard errors. Positive numbers in y-axis indicate later emergence date (differences in emergence dates comparing to current *k* = 0 condition) while negative numbers indicate earlier emregence date. The difference among all DTR conditions are more obvious at the 4^th^ generation than earlier generations.

First we assessed the differences of mean adult emergence date (for generation 1 through 4) between various DTR conditions (*k*≠0) versus the current condition (*k* = 0), when the mean temperature remained the same in each day for all of these conditions (*k*_*1*_ *= k*_*2*_). The results are presented in Fig. 2 (upper panels: Erie, PA; lower panels: Stuarts Draft, VA). When *k* was negative (a smaller than current DTR as *k*<0), the mean emergence date was usually later than current condition (Fig. 2, positive values along Y-axis indicates delayed emergence dates compared to the current condition). This increased number of days to achieve emergence due to changing DTR conditions was consistently larger for the fourth generation than the previous generations, although the pattern of increase with respect to generation differed among locations. When *k* was positive (a larger DTR), the mean emergence date was usually earlier for both locations (Fig. 2, negative values along Y-axis indicated advancing emergence dates compared to the current condition).

**Fig 3:**
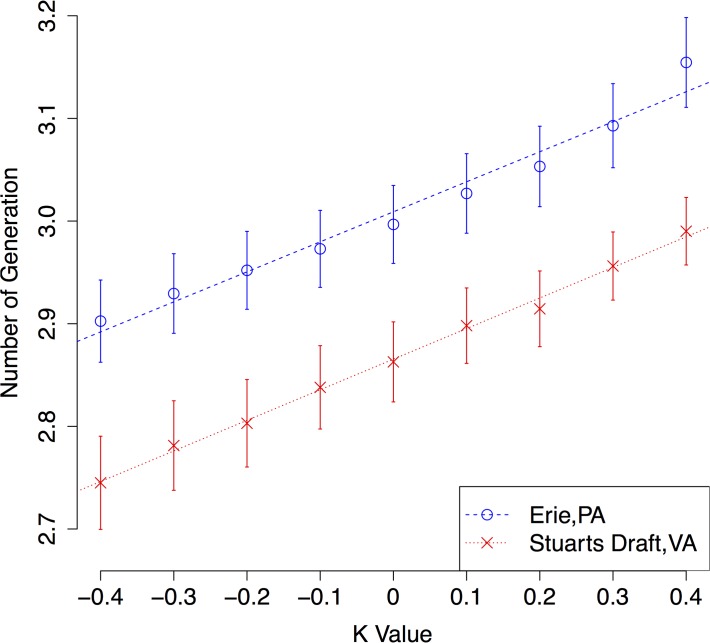
Number of Generations of *P*. *viteana* under Same Mean with Various DTR Change Conditions. Note: The dots represnet mean number of generations (voltinism) with error bars represent standard errors. The lines represent linear regression of voltinism against *k* values for both locations. The slopes of both regression lines are highly significant (*p*<0.01), indicating a strong increasing trend of voltinism with larger DTRs.

The mean number of generations increased in a highly significant linear relationship against *k* for both locations ((Fig. 3, *p*<0.01 from regression results), with Erie and Stuarts Draft experiencing an additional 0.15 and 0.10 generations per year under the largest DTR condition (*k* = 0.4). Our interpretation of *P*. *viteana* voltinism, which defines adults that emerge from overwintering pupae as the first generation, and methods for defining mean voltinism from the realized distribution, are described in S1 Appendix.

The pairwise differences in mean emergence dates among the nine conditions are highly significant (*p*<0.01 from Tukey HSD results) for all the four generations and both locations, with the only exception occurring between the *k* = −0.4 and *k* = −0.3 conditions in the 4^th^ generation (*p* = 0.38 and *p* = 0.15 for Erie and Stuarts Draft, respectively). Thus the emergence dates differ significantly when DTR changed. Mean duration of each generation is summarized in Table 2 for the 9 conditions at both locations. First generation was excluded because it was the overwintering generation from the previous year. Second through fourth generation were counted as complete (egg-to-adult) generations in the current year. A small number of 5^th^ generation *P*. *viteana* did exist, but because of an insufficient number (usually <10), they were also excluded. Regression results indicated consistently highly significant (*p*<0.01) decreasing relationship between DTR and generation duration (i.e. larger DTR was able to shorten generation duration significantly) for all three complete generations in both locations. However the duration of earlier generations (second and third generation) had smaller differences (about 2 days between smallest and largest DTRs), comparing to later generation (about 12 days in Stuarts Draft). This was due to the relatively cooler weather before summer. A simplified explanation for such spatial difference was that Stuarts Draft had larger seasonal temperature fluctuations, and its summer was warmer than Erie, hence the later generations (especially the 4^th^ generation) developed much faster than Erie.

In summary, increasing DTR led to both advancing the mean emergence date and shortening the generation duration (hence increasing the voltinism), even as *Tmean* was held constant.

### Grape Berry Moth Life History Change under Increasing Mean Temperature Conditions (with Various DTRs)

Statistical results of the effects of DTR and *Tmean* changes (through combination of all potential *k*_*1*_ and *k*_*2*_ values) were obtained from the two-way ANOVA with interaction. Both *k*_*1*_ and *k*_*2*_ had highly significant (*p*<0.01) influence on number of generations and mean emergence time for the 2^nd^ through 4^th^ generation for both locations. The interaction between *k*_*1*_ and *k*_*2*_ also highly significantly (*p*<0.01) influence these outputs, with the only exception of the number of generations in Erie (*p* = 0.09). Thus DTR and *Tmean* changes through combination of *k*_*1*_ and *k*_*2*_ can significantly alter *P*. *viteana* phenology.

**Fig 4:**
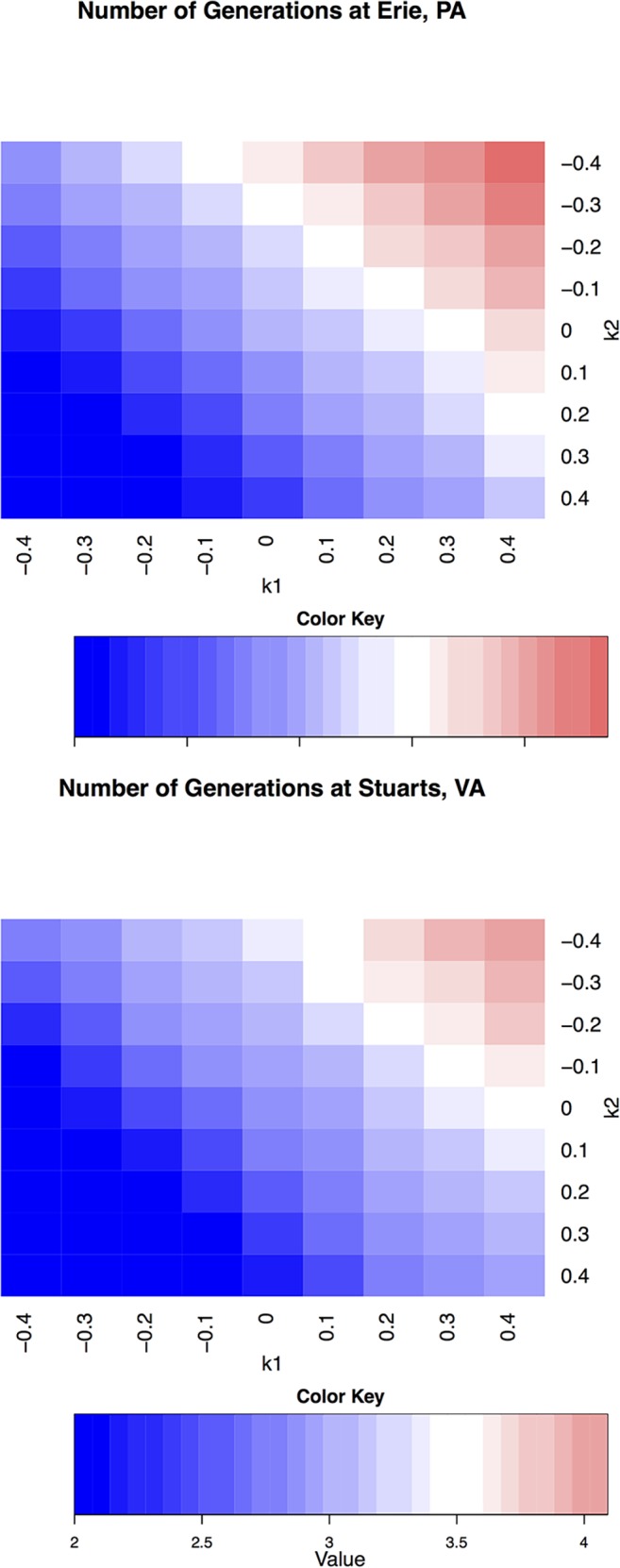
Number of Generations of *P*. *viteana* under Various DTR Change Conditions in Erie, PA and Stuarts Draft, VA. Note: Upper panel for Erie and lower panel for Stuarts Draft. *T’min = Tmin+k*_1_*Tmean and *T’max = Tmax-k*_2_*Tmean. A total of 81 conditions (9 *k*_*1*_×9 *k*_*2*_) are plotted. Cooler color (more blue) indicates lower number of generations and warmer color (more red) indicates higher number of generations. The scales and ranges of color key are the same for Erie and Stuarts Draft, and are directly comparable.

Next, a systematic view of how number of generations of *P*. *viteana* would change under comprehensive climate change conditions is shown in Fig. 4 for Erie and Stuarts Draft, respectively. Current mean voltinism of *P*. *viteana* is about 3 generations per year at both Erie and Stuarts Draft [28]. Under some extreme climate change scenarios, such as *k*_*1*_ = 0.4 and *k*_*2*_ = −0.4 simultaneously (resulting in 4°C increase in *Tmean* and no DTR change—equivalent to shifting the entire diurnal temperature profile, such as the solid black curve in Fig. 1, 4°C upward along the Y-axis), there would be almost 5 generations per year on average, and a few 6^th^ generation *P*. *viteana* adults would be expected as well (see Fig. 3).

The blocks occupying the main diagonal (Fig. 4, from top-left to bottom-right) represent holding the mean temperature conditions the same as in current conditions (a total of 9 blocks where *k*_*1*_ = *k*_*2*_), which is the special case discussed above. In general, the blocks above the main diagonal are associated with increasing mean temperature conditions, and their warmer colors (compared with the diagonal blocks) indicate an increase in voltinism. Similarly the blocks below the main diagonal were linked with decreasing mean temperature conditions. Although decreasing mean temperatures are unlikely, and are not discussed in this study, they are shown for demonstration purposes. Furthermore, the blocks that are in parallel with the main diagonal have the same increased mean temperature (than current condition) but with different DTRs. For example, all five conditions from *k*_*1*_ = 0/*k*_*2*_ = −0.4 to *k*_*1*_ = 0.4/*k*_*2*_ = 0, in parallel with the main diagonal, represent a 2°C increase in *Tmean* for both locations, but with various DTRs. There was a consistent pattern for the same mean temperature conditions (blocks in parallel with main diagonal, regardless if the mean increased or decreased compared to the current condition): larger DTR conditions (towards bottom-right corner) was associated with larger voltinism, for both locations (Fig. 3).

Mild climate change scenarios, such as a 2°C increase in *Tmean*, would impose substantial change in *P*. *viteana* life history between the current condition (*k*_*1*_ = *k*_*2*_ = 0) and projected conditions (Table 3). For example the number of generations increased from 3 (current condition) to approximately 3.7 in Erie, and from 2.9 to 3.5 in Stuarts Draft. Our results indicated that a rarely seen 4^th^ generation of *P*. *viteana* under current climate condition would become a common occurrence by the end of this century. The duration of each generation was also significantly shortened (Table 3).

**Table 3:** Comparison of Number of Generations, Duration of 2^**nd**^-4^**th**^ Generations under Various Climate Change Conditions at Erie, PA and Stuarts Draft, VA.

*k*_*1*_	*k*_*2*_	No. of Generations		Duration of 2^nd^ Gen.		Duration of 3^rd^ Gen.		Duration of 4^th^ Gen.	
0	0	Erie	Stuarts	Erie	Stuarts	Erie	Stuarts	Erie	Stuarts
0	0	3.00	2.87	43.43	49.82	36.78	37.78	28.69	33.95
0	−0.4	3.65	3.38	34.80	44.68	30.46	30.86	24.59	23.19
0.1	−0.3	3.67	3.42	34.83	44.61	30.36	30.67	24.58	22.96
0.2	−0.2	3.70	3.45	34.81	44.54	30.15	30.51	24.44	22.31
0.3	−0.1	3.74	3.49	34.82	44.43	29.89	30.42	24.60	21.96
0.4	0	3.77	3.53	34.96	44.22	29.74	30.21	24.53	21.81

Note: Stuarts stands for Stuart Draft, VA in the table. Annual mean temperature increased by 2°C in rows 2 through 5, but is achieved through different changes in the DTR. A positive *k*_*1*_ increases the *Tmin*. A negative *k*_*2*_ indicates a rising *Tmax*. There is substantial change in mean number of generations and duration of each generation compared to the current condition (row 1, *k*_*1*_ *= k*_*2*_ = 0). Different DTRs also change these life history variables, even with the same 2°C increase in mean temperature. The decimal of number of generations results from taking the average of 1,000 simulations, and the number of generation in each simulation is a discrete integer number.

Additionally, we also observed a noticeable difference of *P*. *viteana* voltinism (and generation duration) among the five different DTR conditions within the same increasing mean temperature scenario (2°C increment in *Tmean*, from *k*_*1*_ = 0/*k*_*2*_ = −0.4 to *k*_*1*_ = 0.4/*k*_*2*_ = 0, in parallel with the main diagonal in Fig. 4). All these conditions resulted in the same 2°C *Tmean* increment but yielded different *P*. *viteana* life histories, such as the number of generations, and mean emergence time (Table 3 and Fig. 4), for both locations. The voltinism difference between the smallest (*k*_*1*_ = 0.4/*k*_*2*_ = 0) and largest (*k*_*1*_ = 0/*k*_*2*_ = −0.4) DTR conditions was about 0.15 generation per year for both locations, similar to the results obtained under the same mean temperature condition. However, the additional 0.15 generations was on top of the 0.5–0.6 increase in number of generations due to changes in mean temperature. The most profound differences were observed in the 4^th^ generation duration in Stuarts Draft (Table 3, last column), where the duration shortened for more than 10 days compared with current conditions, and the largest DTR condition had almost 2 days shorter duration than the smallest DTR condition, while the difference among different DTR conditions in both locations for the 2^nd^ and 3^rd^ generations were all less than 1 day. This result also coincided with the results in previous section (Table 2), where the 4^th^ generation duration had a12-day variability in Stuarts Draft even when *Tmean* was held constant. This demonstrates that temperature changes in Stuarts Draft are due to larger DTR, which accelerated the development of the later generation *P*. *viteana*, especially under larger DTR conditions.

## Discussion

The results of this study demonstrate that changing DTR (with the same or increasing *Tmean*) can substantially change degree-day accumulation and hence, insect life history. The exact patterns depend on the nature of the changes, with effects differing depending on whether there is variation in *Tmax*, *Tmin* or both. Using a previously published degree-day approach we show changes in DTR could impact voltinism, mean generation duration and emergence date above and beyond effects of changes in mean temperatures alone. Indeed, even just altering DTR without altering mean temperature can lead to substantial changes in life history. The effects differ subtly between our study locations indicating interactions with the baseline mean temperature, DTR and photoperiod. The absolute changes in voltinism due to the influence of DTR are small (e.g. 0.10–0.15 generations per year) but because of the ‘compound interest’ effect of population growth, such changes might still have important ecological or economic implications. Interactions with other biotic factors (e.g. phenology shift of host plants and ectotherm predators) could further complicate the life history projection, thus the results of this model still needs validation in the field.

In this study we used 10-year averaged daily temperature data and assumed the DTR change amplitude to be the same across the year. This approach tends to smooth out some extreme conditions. However, extreme climate events (such as abnormally high and low temperatures) [15–16] are expected to increase under future climate change [20,22,36–37] and could well have ecological effects above and beyond the influence of average changes in DTR. Exposure to temperatures above critical maximum temperatures or upper lethal limits, for example, could have dramatic effects on population dynamics, even if the events are rare [38–39].

Our current analysis ignores the potential for local thermal adaptation, which could occur already between populations, or might evolve in the future due to more extreme natural selection under climate change [37,40]. Local adaptation, which could impact base development thresholds or total degree-day accumulation requirements, for example, would alter the response to environmental temperature. How this would affect population dynamics and pest damage is unclear; the nature and extent of local thermal adaptation remains poorly characterized in many systems [40–41]. Similarly, for the grape berry moth, the termination of development is determined by a photoperiod-induced diapause. While photoperiod is fixed for a given day and latitude (i.e. is not going to change in the same way temperature might) local populations could adjust their response to photoperiod in different environments. The Asian tiger mosquito, for example, has shown adaptive evolution of photoperiodic response during invasion and range expansion across a climate gradient in the US [42]. It is possible that *P*. *viteana* might have similar adaptations to optimize its life history in different environmental contexts as well [27].

In summary, our approach uses a basic degree-day model for insect development. Degree-day models are widely used to predict crop, pest and disease emergence and to inform control strategies [28,30,43]. Many of these models do not explicitly include the influence of DTR (at least not in the way we have incorporated it here). Further, many ecological studies exploring possible effects of climate change consider changes in mean temperatures alone. The current study adds to a growing body of literature indicating the need to better quantify the influence of DTR in addition to mean temperatures.

## Supporting Information

S1 Appendix A brief life history modeling procedure of *Paralobesia viteana* voltinism. (DOCX)
