# Supplementary material for: The Influence of Diurnal Temperature Variation on Degree-Day Accumulation and Insect Life History
Source: PLoS One. 2015 Mar 19;10(3):e0120772. doi: 10.1371/journal.pone.0120772 (PMC4366191; doi:10.1371/journal.pone.0120772)
Supplement: S1 Appendix — (DOCX) [file pone.0120772.s001.docx]

**Supporting Information Appendix S1**

**Modeling and Calculation of Grape Berry Moth Voltinism**

The grape berry moth voltinism (number of generations per year) is calculated using an individual-based model that is iterated in a daily time step. At the beginning of each realization (calendar day 1 in the year) of the simulation, an overwintering individual pupa (first generation) is assigned. In summary, three major life history events are modeled in sequence:

1. Temperature-Mediated Diapause Termination: overwintering GBM pupa (first generation only) needs 210 degree-days above a base development threshold (*Tbase*) of 8.4°C to break diapause, develop to adults, and become reproductively mature to initiate oviposition.

2. Temperature-Mediated Development: the successive generations (from second generation on) need a mean of another 424 degree-days to complete the entire life cycle (from egg to reproductively mature adult) under non-diapausing condition (see next events).

3. Photoperiod-Mediated Diapause Induction: the egg stage is critical to determine diapause induction, based on the photoperiod change in a short time window after summer solstice. Thus, the photoperiod experienced by the egg determines whether or not the resulting larva continues to develop to an adult (assuming the larval stages experience sufficient degree days). If the eclosed larva is set to diapause due to declining photoperiod, it develops into a pupa but cannot develop further into adult stage, and thus does not count as a full generation for the current year.

In this model we assume only one viable offspring per individual, thus excluding population size change and focusing on life history. The offspring (successive generations, from second generation and thereafter) loops through the life cycle^1^ until the end of the simulation. By the end of each realization (calendar day 365), only a discrete integer number of adults (the maximum possible generation of generations) resulting from each initialized overwintered pupa is reported (e.g. 2, 3, 4, or 5). Meanwhile, the emergence date of each generation’s adult is also recorded. By simulating 1,000 realizations we collect 1,000 integers at the end of the entire simulation, representing the *distribution* of number of generations (voltinism). The mean voltinism is the arithmetic average of these 1,000 integers. For instance, the current mean voltinism in Stuart is 2.87. The fraction comes from taking the mean of a population of discrete 2,3,4 (and possibly 5) generation adults and should not be interpreted as a single realization producing a fractional voltinism. Consequently, although the mean voltinism in Stuart is below 3, it is not impossible for some realizations to produce 4 or even 5 generations adults per year. Please note that although the first generation adult develops from the overwintering pupa from the previous year, and does not develop from egg to adult within the current year (and it does not cause direct economic damage, since only larva is harmful to the vineyard), it is still included as a generation to calculate the voltinism because it terminates diapause and develops into adult, and this is consistent with existing literature.
